# Unexpected diagnosis: large hemangioma in the interatrial septum

**DOI:** 10.1186/s13019-024-02794-9

**Published:** 2024-05-30

**Authors:** Begoña Bernal-Gallego, Verónica Hernández-Jiménez, Lidia Castillo, Rosa González-Davia, Nieves De Antonio-Antón, Guillermo Reyes-Copa

**Affiliations:** 1 Cardiothoracic Surgery Department, Hospital Universitario de la Princesa, Calle de Diego de León, 62, Madrid, Span, 28006 Spain; 2 Cardiology Department, Hospital Infanta Cristina, Av. 9 de Junio, 2, Parla, Madrid, 28981 Spain; 3 Histopathology Department Hospital Universitario de la Princesa, Calle de Diego de León, 62, Madrid, 28006 Spain

**Keywords:** Cardiac hemangioma, Interatrial septum, Histopathological examination, Echocardiogram, Case report

## Abstract

**Background:**

Cardiac hemangiomas are very uncommon benign primary tumors. They are usually located preferentially in the right atrium and their location in the interatrial septum is extremely rare.

**Case presentation:**

We report the case of a 41-year-old patient who was admitted due to a stroke. The transthoracic echocardiogram revealed a large mass in the right atrium adhered to the interatrial septum. Suspecting an atrial myxoma, surgical intervention was performed confirming that the mass extended within the thickness of the interatrial septum, protruding into the right atrial cavity. The histologic report confirmed a hemangioma.

**Conclusions:**

Cardiac hemangiomas are rare primary tumors and are usually misdiagnosed as other cardiac tumors. Histopathological examination is essential for a definitive diagnosis.

## Background

Cardiac hemangiomas are very rare benign primary tumors [1]. Most of the time, they are incidental findings during autopsies or routine examinations. They are usually located preferentially in the right atrium (RA) [2]. We present the case of a young patient with a hemangioma located in the interatrial septum (IAS).

## Case presentation

A 41-year-old man, an active smoker who was previously asymptomatic, presented to the emergency room with symptoms of hemiparesis and hypoesthesia that had lasted for 8 h. The electrocardiogram showed sinus rhythm, and the head computed tomography (CT) scan did not reveal any acute cranial pathology. Due to the persistence of symptoms, intravenous fibrinolysis was performed. Subsequently, the patient remained asymptomatic, and no bleeding was observed in the follow-up CT scan.

**Fig. 1:**
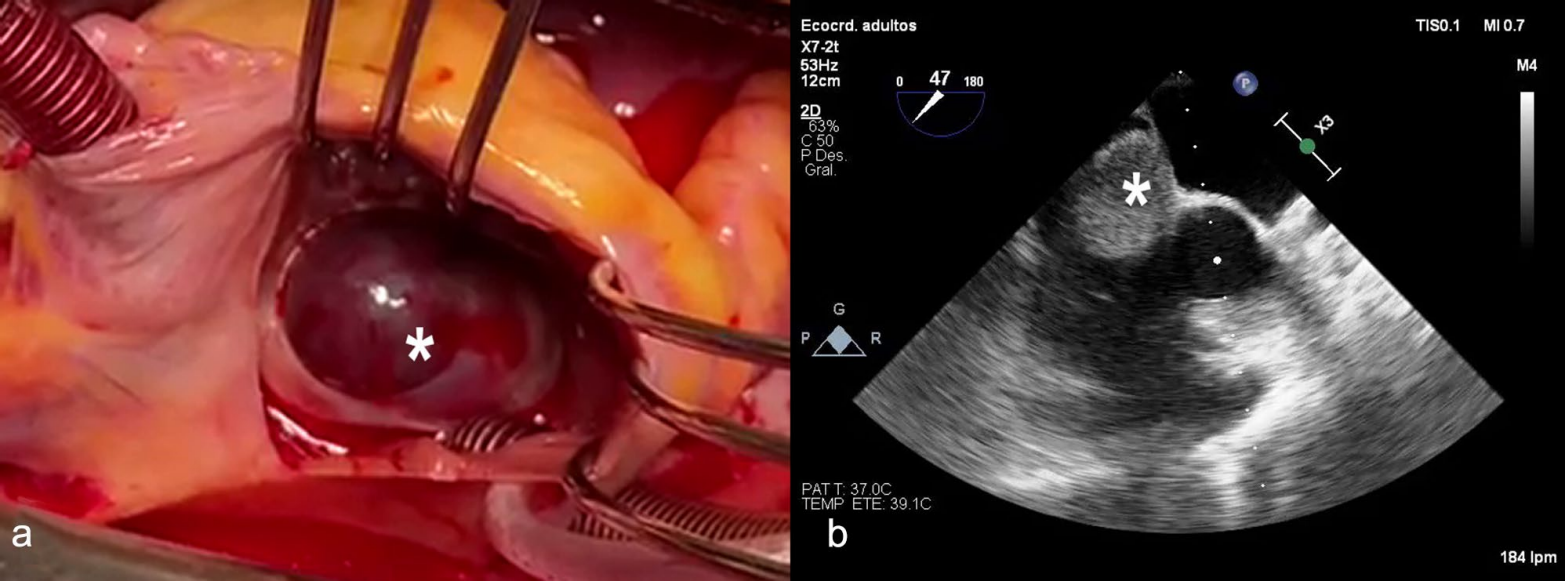
(**a**) *Intraoperative hemangioma. Surgical vision from the AR. (**b**) Transthoracic echocardiogram showing the mass in the RA adhered to the IAS

In the investigation of stroke in a young patient, a transthoracic echocardiogram (TTE) was performed, which revealed a mass in the RA adhered to the IAS. Left and right ventricular function were normal, and no valvulopathies were detected. The transesophageal echocardiogram showed a large, well-defined, echodense mass measuring 4.77 cm x 2.51 cm prolapsing into the RA cavity, with a broad implantation base in the IAS, and close to the superior vena cava (Fig. 1). Due to the suspicion of an atrial myxoma, the patient was transferred to our center and surgery was performed. A median sternotomy and aortobicaval cannulation were performed. A right atriotomy revealed a solid and well-defined tumor measuring 4 × 3 cm within the thickness of the IAS, protruding into the right atrial cavity (Fig. 1). No patent foramen ovale was observed. The tumor was completely resected. As a complication, the patient developed a complete atrioventricular block during the surgery and the recovery period which required the implantation first of a temporary epicardic pacemaker and then a permanent pacemaker. It is probably due to damage to the conduction system from excision of the tumor located close to the superior vena cava. A postoperative transthoracic echocardiogram was performed without evidence of any recidivating masses and the patient was discharged home on the 12th postoperative day.

**Fig. 2:**
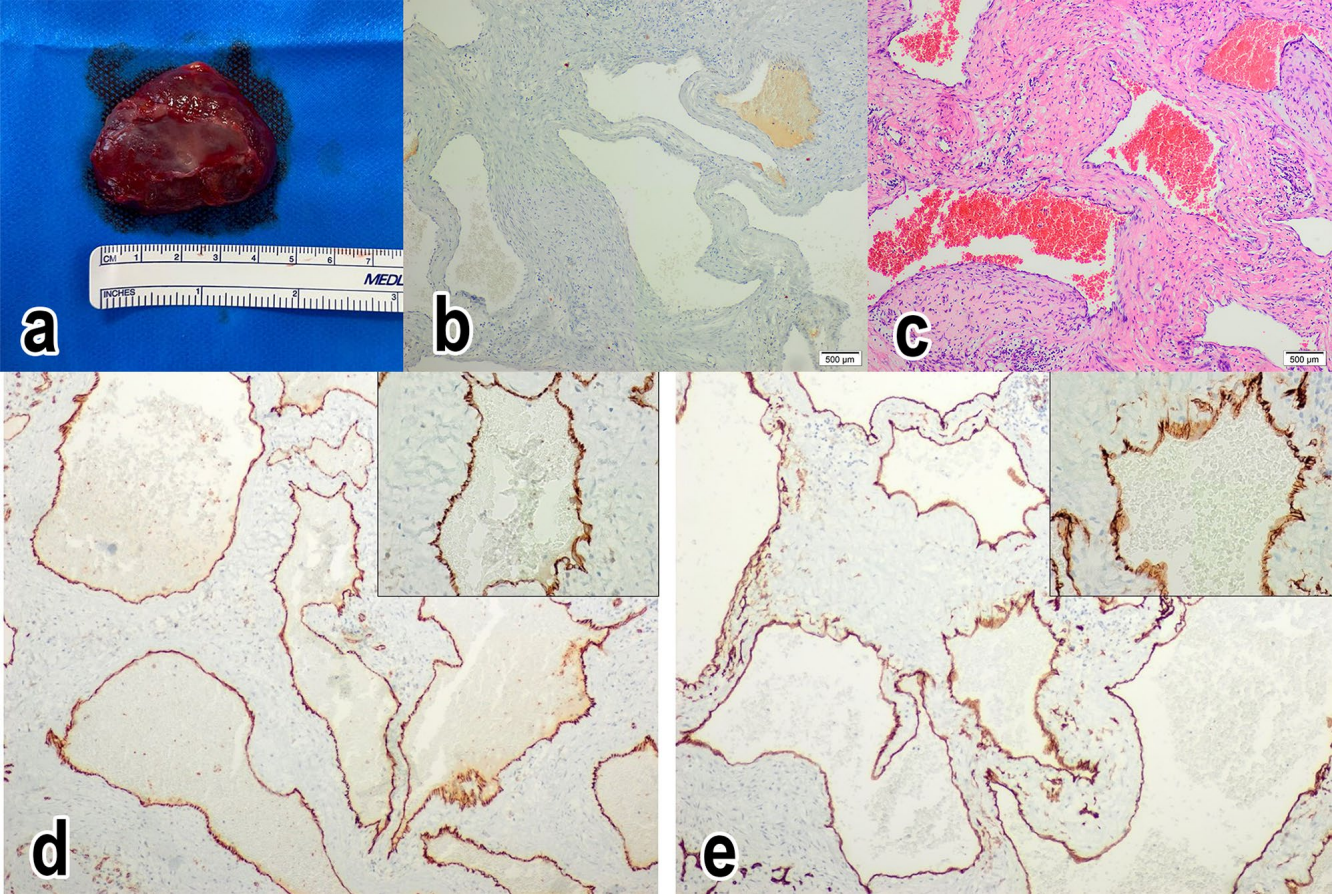
(**a**) Intraoperative specimen removed. (**b**) Histologic appearance of the hemangioma: negative calretinin stain. Magnification is 10X. (**c**) Hematoxylin and eosin stain. Magnification is 10X. (**d**) Immunohistochemical staining shows positive expression of CD31 antibodies in the tumoral endothelium. Magnification is 10X and 40X. (**e**) Immunohistochemical staining shows positive expression of CD34 antibodies in the tumoral endothelium. Magnification is 10X and 40X

The histological examination of the mass revealed vascular proliferation of capillaries sized vassels anastomosed with each other, covered by flat epithelium without cytological atypia. Between the vascular structures, connective tissue, few inflammatory infiltrates and some intermediate-sized cells with clear cytoplasm are observed. The Immunohistochemical staining shows positive expression of CD34 and CD31 antibodies in the tumoral endothelium. Calretinin staining was negative. The definitive histological diagnosis was hemangioma (Fig. 2).

At 6 months after the intervention, the patient remained asymptomatic, and follow-up TTE did not show any signs of tumor recurrence.

## Discussion

Cardiac hemangiomas are rare primary tumors, accounting for approximately 1–2% of primary cardiac tumors [1]. These tumors can occur at any age and in any location, but they are mostly found in the left atrium and left ventricle, and extremely rare in the IAS, as in the case we present [2]. They usually measure between 20 and 50 mm. Most patients with cardiac hemangiomas are asymptomatic and are incidentally found during routine examinations or autopsies. The symptoms will depend on the location, size, and growth rate [3]. Left atrial hemangiomas usually cause dyspnea, intolerance to stress, angina, or systemic embolism, while right atrial or right ventricular hemangiomas can lead to systemic congestion. Intramural or septal hemangiomas can invade the conduction system and cause atrioventricular block and sudden death [2, 4].

Transthoracic echocardiography is the preferred technique for the initial diagnosis of intracardiac masses due to its safety, noninvasiveness, and low cost. Cardiac CT or MRI can provide more precise characterization of the dimensions, location, and degree of infiltration of the mass. In the case of cardiac hemangiomas, coronary arteriography can be useful to determine the coronary distribution, the vessels that feed the tumor, or the possible compression of the coronary arteries [5].

The differential diagnosis should include thrombi or other cardiac tumors, such as myxoma. In fact, preoperative diagnostic errors are common, as in our case. Histopathological examination is essential for a definitive diagnosis.

Regarding the management of cardiac hemangiomas, there is controversy, but most centers agree that surgical removal is the treatment of choice, especially in symptomatic cases [6]. After complete resection, the prognosis is generally favorable, with a low recurrence rate. Furthermore, incomplete resection can also lead to long-term survival benefits [7].

## Conclusions

Cardiac hemangioma is a rare, slow-growing, benign tumor, and its location in the interatrial septum is extremely rare. Echocardiography is a fundamental diagnostic technique, providing a precise method for diagnosis, which requires histopathological examination for confirmation. Surgical resection is the treatment of choice, and the prognosis is usually favorable. In our case, the patient had a satisfactory recovery after resection and is being followed up without any signs of recurrence.

## Data Availability

Not applicable.

## Abbreviations

RA: Right atrium
IAS: Interatrial septum
CT: Computed tomography
TTE: Transthoracic echocardiogram
MRI: Magnetic resonance image
